# Automated region growing-based segmentation for trabecular bone structure in fresh-frozen human wrist specimens

**DOI:** 10.1186/s12880-024-01281-w

**Published:** 2024-05-01

**Authors:** Eva Klintström, Benjamin Klintström, Örjan Smedby, Rodrigo Moreno

**Affiliations:** 1 Center for Medical Image Science and Visualization (CMIV), Linköping University, Linköping, SE-58185 Sweden; 2 Department of Radiology and Department of Health, Medicine and Caring Sciences, Linköping University, Linköping, SE-58185 Sweden; 3 Department of Biomedical Engineering and Health Systems, KTH Royal Institute of Technology, Hälsovägen 11C, Huddinge, SE-14157 Sweden

**Keywords:** Trabecular Bone, Bone Structure Analysis, Segmentation, Micro-CT

## Abstract

Bone strength depends on both mineral content and bone structure. Measurements of bone microstructure on specimens can be performed by micro-CT. In vivo measurements are reliably performed by high-resolution peripheral computed tomography (HR-pQCT) using dedicated software. In previous studies from our research group, trabecular bone properties on CT data of defatted specimens from many different CT devices have been analyzed using an Automated Region Growing (ARG) algorithm-based code, showing strong correlations to micro-CT.

The aim of the study was to validate the possibility of segmenting and measuring trabecular bone structure from clinical CT data of fresh-frozen human wrist specimens. Data from micro-CT was used as reference. The hypothesis was that the ARG-based in-house built software could be used for such measurements.

HR-pQCT image data at two resolutions (61 and 82 µm isotropic voxels) from 23 fresh-frozen human forearms were analyzed. Correlations to micro-CT were strong, varying from 0.72 to 0.99 for all parameters except trabecular termini and nodes. The bone volume fraction had correlations varying from 0.95 to 0.98 but was overestimated compared to micro-CT, especially at the lower resolution. Trabecular separation and spacing were the most stable parameters with correlations at 0.80-0.97 and mean values in the same range as micro-CT.

Results from this in vitro study show that an ARG-based software could be used for segmenting and measuring 3D trabecular bone structure from clinical CT data of fresh-frozen human wrist specimens using micro-CT data as reference. Over-and underestimation of several of the bone structure parameters must however be taken into account.

## Introduction

Bone strength is determined by bone mineral content as well as the trabecular and cortical bone microstructure. The mineral content can be measured by volumetric Dual-energy X-ray Absorptiometry (DXA). This however, only reflects about 60-70% of the variation in bone strength [1].

Measurements of trabecular and cortical bone microstructure can reliably be performed by invasive bone biopsies and analyzed by 2D histological sections or by 3D micro-computed tomography (micro-CT) [2]. With high-resolution peripheral quantitative CT (HR-pQCT), it is possible to visualize bone cortical and trabecular microstructure of the peripheral skeleton, e.g., the radius and tibia, in vivo [3, 4]. Multi-slice CT (MSCT) devices are used in vivo for diagnostic imaging of the whole human body. Another technique that has been used in many in vitro and a few in vivo studies for measurements of trabecular bone structure is cone beam CT (CBCT) [5, 6].

To be able to compare the capability of different devices for bone microstructure analysis, it is of great importance to use standardized nomenclature and units [7, 8]. The analyses depend on the segmentation methods, where many different techniques exist [9]. Various software tools are available for the calculation of trabecular bone structure parameters from high spatial and high contrast-to-noise resolution (CNR) image data sets like in micro-CT [10]. Segmentation of image data from clinical devices, which all have lower spatial resolution and CNR is more demanding especially due to the partial voxel effect. HR-pQCT data can be segmented, and the bone structure can be analyzed using dedicated software from the manufacturer [4]. To the best of our knowledge, no bone segmentation software dedicated and validated for MSCT or dental CBCT devices exists. Still, in experimental studies, different in-house developed software have been evaluated [5, 11, 12] for use with MSCT, CBCT as well as with HR-pQCT data.

So far, our in-house developed code, based on the Automated Region Growing (ARG) algorithm [13], has been used for segmenting and analyzing imaging data of small defatted cubic radius bone specimens imaged in micro-CT, dental CBCT and MSCT using different imaging parameters. Strong correlations were found between scanners intended for in vivo use and the reference method micro-CT [5, 14, 15]. Regarding HR-pQCT data, our software showed promising results for bone structure parameters like bone volume, trabecular nodes, separation, spacing, number and thickness, with correlations to micro-CT varying between 0.72 to 0.93. When analyzing tiny structures like termini and nodes, the ARG-based code was superior to the HR-pQCT dedicated software, but somewhat inferior for other parameters like trabecular thickness and number. [5]. It would be of interest to study if this segmentation code could also be applicable for imaging data of fresh-frozen radius bone specimens.

An advantage of this ARG-based segmentation software is its possibility to segment and analyze data from many different clinical modalities like CBCT, EIDCT (energy-integrated detector CT) and PCDCT (photon-counting detector CT) for which, now, there are no dedicated software’s available.

## Aim

The aim of the study was to validate the possibility of using an ARG-based segmentation code for segmenting trabecular bone microstructures (TBMS) and measuring TBMS parameters (such as BV/TV) of fresh-frozen human wrist specimens scanned using HR-PQCT, and to compare this data with that of micro-CT. The hypothesis was that the trabecular bone microstructures of human wrist can be segmented, and the parameters can be measured when scanned using HR-pQCT.

## Material and Method

### Material

Image data from 23 fresh-frozen forearms were included in the study. The donors were 7 males and 5 females with a mean age of 77 years (standard deviation of 9 years and range 65 to 92 years) who, after informed consent, had donated their bodies to research. Ethical approval was obtained by the Medical University of Vienna [16]. As described in a previous study, the forearms were stored at room temperature before scanning in HR-pQCT [17]. The device was an XtremeCTII (SCANCO Medical AG, Brüttisellen, Switzerland). Two different protocols were used, one at low resolution (LR) 82 μm isotropic voxels, 68kV, 1460μA, 36ms, and the other at high resolution (HR) 61μm isotropic voxels 68kV, 1460μA, 43ms. According to the recommendation from the manufacturer for the second-generation HR-pQCT devices, the starting point for the scans is 9 mm from the endpoint of the distal radius plafond. The scanned and analysed sections of each forearm are 20 mm long. The forearms were repositioned and rescanned three times for reproducibility reasons. Before scanning in micro-CT μCT100 (SCANCO Medical AG, Brüttisellen, Switzerland) at 16.4μm, voxels, 70kV, 200μA, 300s, the 20mm sections were cut off using a diamond-coated band saw, and the bone marrow was removed. Detailed information on this procedure can be found in [16].

### Data processing, segmentation, and analysis of trabecular bone structure parameters

**Fig. 1:**
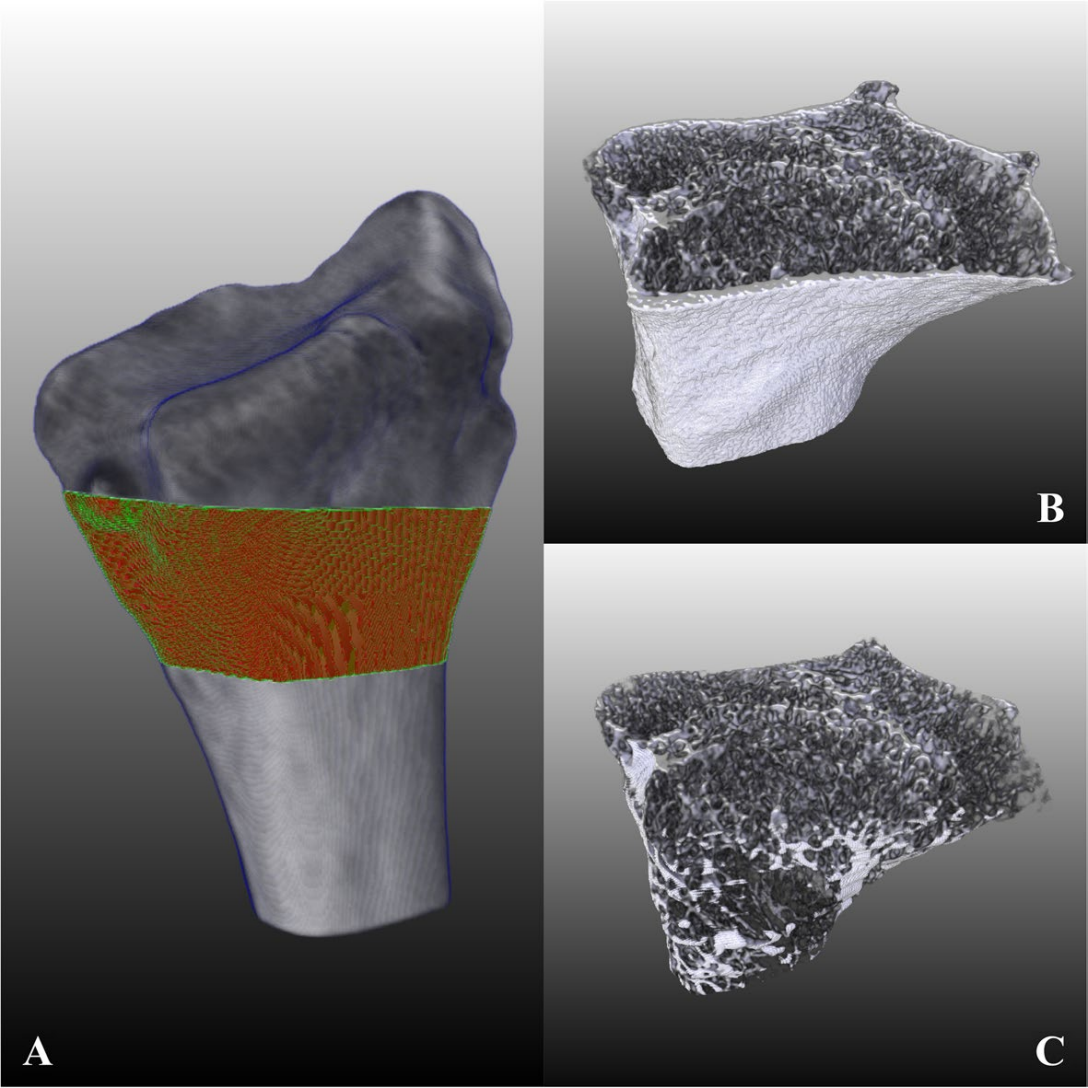
Images of the radius for demonstration of the analysed volumes, where **A** shows the placement of the analysed volume; **B** the volume including cortical bone; **C** the analysed trabecular volume

**Fig. 2:**
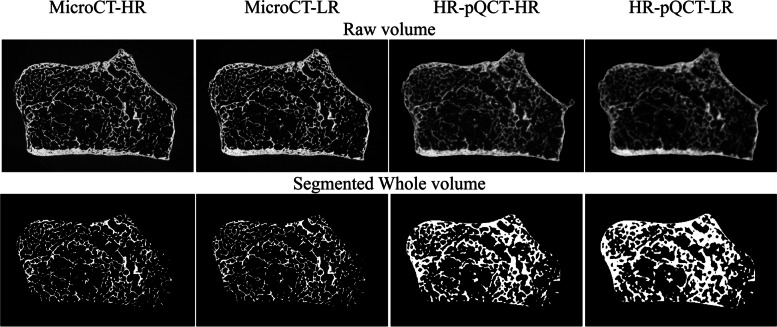
Images slices of the radius. Columns: Micro-CT high resolution (HR) (16.4µm), Micro-CT low resolution (LR) (32.8µm), HR-pQCT HR (61µm) and HR-pQCT LR (82µm). Rows: upper row, raw image slices; lower row, segmented trabecular volumes

All analyses are in 3D after registration of the volumes to each other using the automatic 3D spatial rigid registration method of the elastic software as described in [16]. Masks for removing the cortical bone from the 23 specimens were created manually in MeVisLab (MeVis Medical Solutions AG, Bremen, Germany). Initially, there were 24 specimens available for analysis. Due to limitations in the method used for creating the masks, one specimen had to be excluded, resulting in 23 specimens available for further analysis. The analysed, trabecular volumes of the wrists can be seen in Fig. 1 and Fig. 2. The trabecular bone data sets of the micro-CT were down-sampled to a voxel size of 32.8µm to reduce the memory requirements. The elapsed time for segmenting and analysing one data set with 32.8µm voxels varies depending on the bone structure in the specimen but is about 5-10 minutes. A volume with voxels of 61 or 82 µm could be analysed in about 2-3 minutes.

The ARG algorithm, based on homogeneity, is used to separate bone from bone marrow (background) in the volumes and to obtain binary images [13, 14]. The method starts with a very limited homogeneity threshold, resulting in under-segmentation. The processing is repeated with higher thresholds and iterated. The iteration where both the bone and the background regions reached the highest homogeneity is used to analyse the following bone structure parameters:

- Bone Volume over Total Volume (BVTV): the number of voxels in the analysed volume segmented as bone divided by the total number of voxels in the volume of interest.
- Trabecular Thickness (Tb.Th): the mean width of trabeculae in mm
- Trabecular Spacing (Tb.Sc): the mean of the minimum distance between the midlines of neighbouring trabeculae in mm.
- Trabecular Separation (Tb.Sp): the mean of the minimum distance between the edges of neighbouring trabeculae in mm
- Trabecular Nodes (Tb.Nd): the number of intersections of the skeleton per mm^3^.
- Trabecular termini (Tb.Tm): the number of free ends per mm^3^

The method for calculating Tb.Th, Tb.Sc and Tb.Sp can be used to create 3D local maps [18]. As the values of those parameters can vary within each volume, their standard deviation (s) within the volume was also calculated as a measure of dispersion.

The contrast-to-noise (CNR) ratio (unitless) was measured to evaluate potential differences in noise between the devices. The CNR was calculated as the difference in mean intensity between the foreground (skeletonized bone) and the background (bone marrow for HR-pQCT and saline water for micro-CT) divided by the standard deviation of the background.

The micro-CT data could be segmented using Otsu-thresholding [19] due to high contrast images and a low number of partial voxels (upper row in Fig. 2). The computer used for the calculations was a Linux desktop (Ubuntu 20.04.4 LTS) with an AMD Ryzen 9 5900x and 128GB RAM. The micro-CT data for the whole trabecular bone volume measurements were down-sampled from 16.4µm voxels to 32.8μm voxels to reduce the processing time.

In summary, nine bone structure parameters were computed: BVTV, Tb.Th, Tb.Sc, Tb.Sp, Tb.Nd, Tb.Tm as well as the volume-based standard deviations s(Tb.Th), s(Tb.Sc) and s(Tb.Sp).

### Statistical analysis

Mean values with standard deviations were calculated for the nine bone structure parameters. To study the linear relationship between structure parameters obtained from HR-pQCT and those obtained from micro-CT, we used Matlab version R2020a Update 3 (Mathworks, Natick, Massachusetts, USA) to calculate coefficients of determination (*R*^2^), Pearson linear correlations with 95% confidence intervals as well as *p *values for the null hypothesis *r*=0. To evaluate systematic over- and underestimation, Bland-Altman plots with 95% limits of agreement were constructed for structure parameters obtained from HR-pQCT and from micro-CT.

## Results

The data did not significantly deviate from the normal distribution.

**Fig. 3:**
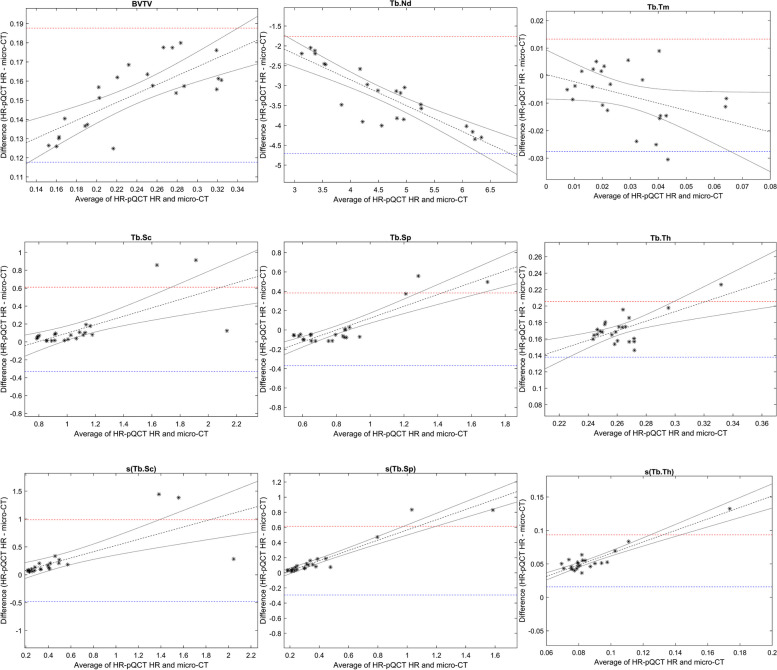
Bland Altman plots for micro-CT and HR-pQCT (HR). Micro-CT voxel size 32.8µm and HR 61µm. Upper and lower 95% limits of agreement are shown as dashed lines in red and blue, respectively: linear regression with 95% confidence limits in black

**Fig. 4:**
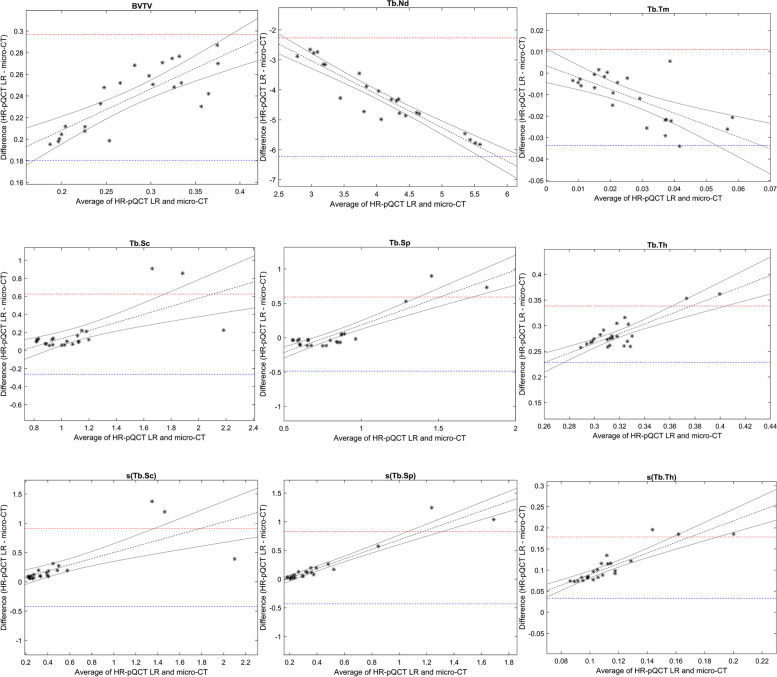
Bland Altman plots for micro-CT and HR-pQCT (LR) Micro-CT voxel size 32.8µm and LR 82µm Upper and lower 95% limits of agreement are shown as dashed lines in red and blue, respectively: linear regression with 95% confidence limits in black

Strong correlations were found between micro-CT and HR-pQCT regarding all trabecular bone structure parameters. Correlation coefficients varied between 0.76 and 0.98 for the HR (voxel size 61µm) and 0.59 and 0.96 for the LR (voxel size 81µm) protocol (Table 1). Bland Altman analysis indicated an overestimation of BVTV, Tb.Th and s(Tb.Th) in all cases increased with the measured values, and underestimation of Tb.Nd, also most pronounced at larger measured values (Figs. 3 and 4)

**Table 1:** Pearson linear correlation with micro-CT: coefficients with 95% confidence intervals for the 3D bone structure parameters

		HR-pQCT-HR(61µm)	HR-pQCT-LR(82µm)
Average measures	BVTV	**0.98(0.95;0.99)**	**0.96(0.90;0.98)**
	Tb.Th	0.76(0.50;0.89)	0.72(0.44;0.88)
	Tb.Sc	0.87(0.71;0.94)	0.88(0.74;0.95)
	Tb.Sp	**0.93(0.84;0.97)**	0.90(0.77;0.95)
	Tb.Nd	0.91(0.80;0.96)	0.85(0.67;0.93)
	Tb.Tm	0.83(0.64;0.93)	0.83(0.63;0.92)
Dispersion measures	s(Tb.Th)	**0.91(0.81;0.96)**	0.59(0.23;0.8)
	s(Tb.Sc)	0.84(0.65;0.93)	0.87(0.71;0.94)
	s(Tb.Sp)	**0.97(0.93;0.99)**	**0.95(0.88;0.98)**

Bold figures represent correlations > 0.9

*HR-pQCT* High Resolution peripheral Quantitative Computed Tomography, *BVTV* Total bone volume, *Tb.Th* Trabecular thickness, *Tb.Sc* Trabecular spacing, *Tb.Sp* Trabecular separation, *Tb.Nd* Trabecular nodes, *Tb.Tm* Trabecular termini, s(Tb.Th), s(Tb.Sc) and s(Tb.Sp) are the intra-volume standard deviation for Tb.Th, Tb.Sc and Tb.Sp, respectively

**Fig. 5:**
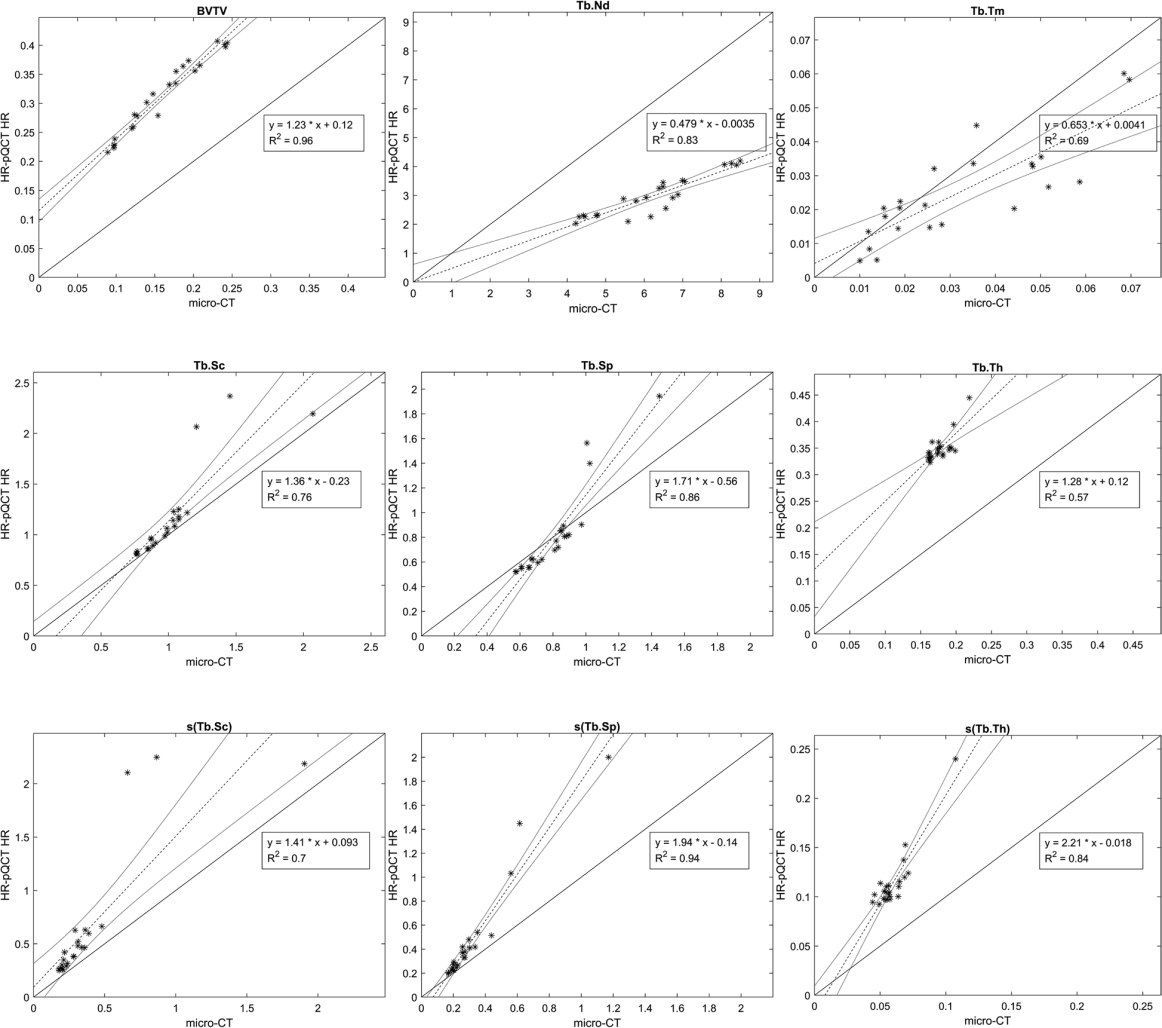
Scatter plots with coefficients of determinations (R^2^) for micro-CT and HR-pQCT (HR) . Micro-CT voxel size 32.8µm and HR 61µm

**Fig. 6:**
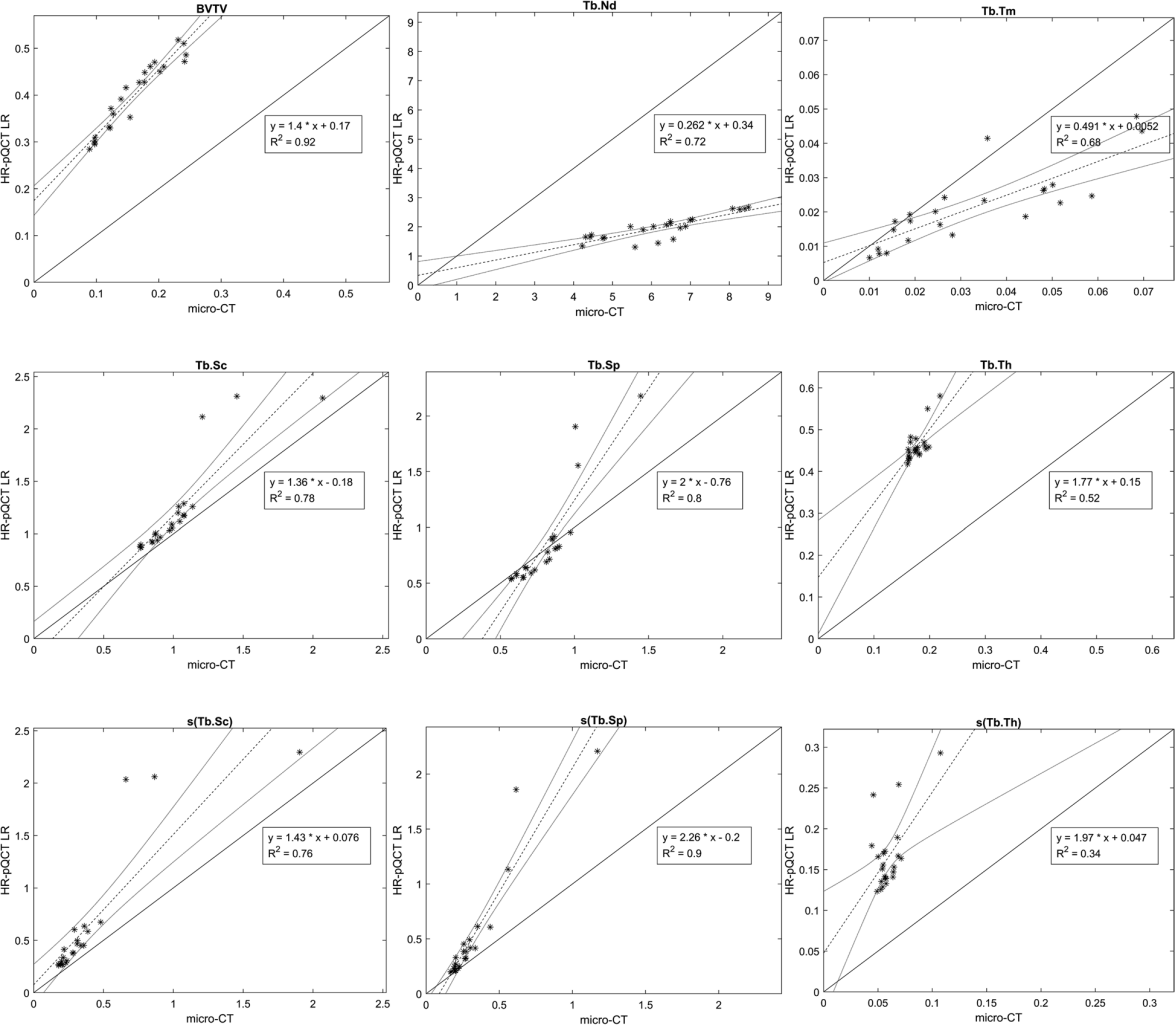
Scatter plots with coefficients of determinations (R^2^) for micro-CT and HR-pQCT (LR). Micro-CT voxel size 32.8µm and LR 82µm

BV/TV and Tb.Th were overestimated about three times when comparing LR to micro-CT. The HR data overestimated BV/TV and Tb.Th about two times. Tb.Sc and Tb.Sp were more consistent and less affected by alteration in voxel size. (Table 2 and Fig. 5). Regarding average measures and dispersion measures of Tb.Th, Tb.Sp and Tb.Sc, the measures from the HR-pQCT data (both LR and HR) did not significantly differ from micro-CT (Table 3). The same trends of over- and underestimation could be visualized in the descriptive statistics, the scatter plots and the Bland Altman plots (Table 2, Figs. 3, 4, 5 and 6).

**Table 2:** Descriptive statistics for nine 3D bone structure parameters and CNR

	Bone structure	Micro-CT(32.8µm)	HR-pQCT-HR(61µm)	HR-pQCT-LR(82µm)
Average measures	BVTV	0.16±0.05	0.31±0.06	0.4±0.08
	Tb.Th	0.18±0.02	0.35±0.03	0.46±0.04
	Tb.Sc	1.02±0.28	1.16±0.44	1.20±0.43
	Tb.Sp	0.81±0.19	0.82±0.36	0.86±0.44
	Tb.Nd	6.21±1.34	2.97±0.7	1.96±0.41
	Tb.Tm	0.03±0.02	0.03±0.01	0.02±0.01
	CNR	12.57±1.91	6.98±0.74	6.68±0.73
Dispersion measures	s(Tb.Th)	0.06±0.01	0.11±0.03	0.17±0.04
	s(Tb.Sc)	0.39±0.37	0.64±0.62	0.63±0.61
	s(Tb.Sp)	0.32±0.22	0.48±0.44	0.52±0.52

Data are presented as mean± standard deviation

*HR-pQCT* High Resolution peripheral Quantitative Computed Tomography, *BVTV* Total bone volume, *Tb.Th* Trabecular thickness, *Tb.Sc* Trabecular spacing, *Tb.Sp* Trabecular separation, *Tb.Nd* Trabecular nodes, *Tb.Tm* Trabecular termini, s(Tb.Th), s(Tb.Sc) and s(Tb.Sp) are the intra-volume standard deviation for Tb.Th, Tb.Sc and Tb.Sp, respectively

**Table 3:** T-test for difference between scanners presented as p-value (95%-confidence intervals of the difference)

		HR-pQCT-HR(61µm)	***HR-pQCT-LR(82µm)***
Average measures	BVTV	***p*<0.0001(-0.19;-0.12)**	***p*<0.0001(-0.28;-0.2)**
	Tb.Th	***p*<0.0001(-0.18;-0.16)**	***p*<0.0001(-0.3;-0.27)**
	Tb.Sc	*p*=0.205(-0.36;0.08)	*p*=0.0998(-0.4;0.04)
	Tb.Sp	*p*=0.9332(-0.18;0.17)	*p*=0.604(-0.26;0.15)
	Tb.Nd	***p*<0.0001(2.6;3.88)**	***p*<0.0001(3.65;4.84)**
	Tb.Tm	*p*=0.1555(0;0.02)	***p*=0.017(0;0.02)**
Dispersion measures	s(Tb.Th)	***p*<0.0001(-0.07;-0.04)**	***p*<0.0001(-0.13;-0.09)**
	s(Tb.Sc)	*p*=0.1019(-0.56;0.05)	*p*=0.1092(-0.54;0.06)
	s(Tb.Sp)	*p*=0.1245(-0.37;0.05)	*p*=0.0987(-0.44;0.04)

Bold figures represent data with *p*<0.05

*HR-pQCT* High Resolution peripheral Quantitative Computed Tomography, *BVTV* Total bone volume, *Tb.Th* Trabecular thickness, *Tb.Sc* Trabecular spacing, *Tb.Sp* Trabecular separation, *Tb.Nd* Trabecular nodes, *Tb.Tm* Trabecular termini, s(Tb.Th), s(Tb.Sc) and s(Tb.Sp) are the intra-volume standard deviation for Tb.Th, Tb.Sc and Tb.Sp, respectively

The contrast-to-noise ratio (CNR) varied between 6.9 and 12.6, with the highest value for the micro-CT data. The lowest CNR was measured for the LR data (Table 2).

## Discussion

For the bone research community, it would be of great interest to develop a segmentation code that is useful for trabecular bone structure analysis of data from clinical CT-devices. This applies especially to those devices, like CBCT, that lack trustworthy HU values. If data from a validated CT-device, like HR-pQCT, could be analyzed with strong correlation and agreement with the reference method of micro-CT, that would be a step towards reaching that goal.

In this *in-vitro* study, 23 human radius specimens were examined by micro-CT (16.4µm voxel size down-sampled to 32.8µm) and HR-pQCT (61 and 82µm voxel size). We found strong correlations between trabecular bone 3D microstructure parameters extracted from HR-pQCT and micro-CT when using our in-house ARG-based segmentation code for segmenting this data.

There were significant differences in mean values between micro-CT and HR-pQCT using our software for the analyses. One of the most stable parameters was Tb.Sp. This is in agreement with an earlier study comparing three brands of micro-CT scanners at different spatial resolutions [20]. In another earlier published study, four different software tools were used to analyse 701 segmented micro-CT images of bone samples from the radius, femur, and spine [10]. Although the samples in that study were scanned with the same micro-CT using the same scanning parameters there were differences in the mean values, whereas the correlations were strong. The differences varied depending on which parameters were studied and which software packages were compared. In yet another study, HR-pQCT data at 60 µm and micro-CT data at 20 µm resolution from wrist and trapezia were analysed with two commercial software packages. The only one of the five different parameters analysed by the two software, that could directly be compared was the bone volume fraction (BVTV) [21]. The above-mentioned studies indicate the need for establishing universal standards and segmentation methods to facilitate comparison between image data from different clinical CT devices.

In this study, mean values for Tb.Th and BV/TV were overestimated when comparing micro-CT and LR HR-pQCT at 82µm voxels (Table 2). Comparing the LR (82µm voxels) to the HR (61µm voxels) data resulted in less pronounced overestimations. The degree of overestimation seems strongly related to differences in voxel sizes. The partial volume effect, related to voxels partially consisting of bone and partially of bone marrow, negatively influences the analyses of bone parameters. Another factor having a negative impact on the analyses of bone structure parameters is CNR, which is lower in HR-pQCT compared to micro-CT. The parameter most sensitive to changes in resolution is Tb.Tm. This could be related to the fact that small irregularities in the trabecular surface will be registered as termini when the resolution is high. More research and evaluation of the software are needed before including Tb.Tm in future analyses. The ARG-based segmentation code used in this study has previously been tested in earlier studies on defatted trabecular bone cubic specimens. The correlations regarding bone structure parameters between micro-CT and the analysed CT devices (CBCT, HR-pQCT, EIDCT and PCDCT) have been strong [5, 15, 22, 24]. In this study, the segmentation code was also shown to be useful for analysing trabecular bone structures from datasets of whole fresh-frozen radius specimens.

A limitation of this study is the time needed for analyses of micro-CT data with high resolution, resulting in large datasets. In this study, we therefore down-sampled the micro-CT from 16.4µm to 32.8µm. Another limitation is the overestimation of the trabecular thickness and total bone volume. The CT technique is constantly evolving, with photon counting detector CT (PCDCT) being one of the most recent developments. In a recent i*n vitro* study, it is shown that trabecular bone 3D structure can be analysed with a strong correlation to the clinical gold standard of HR-pQCT [23]. In another recently published in-vitro study, PCDCT showed strong correlations to micro-CT regarding trabecular bone structure parameters [24]. But even with this technique, overestimation of bone volume seems hard to overcome. Although the resolution is higher than that of conventional energy-integrated detector CT scanners, the resolution for PCDCT is still near the actual trabecular thickness.

Resolution close to the thickness of trabecular bone structures results in voxels partially consisting of bone and partially bone marrow, which has a negative effect on the segmentation with an overestimation of the bone volume fraction. The problem with bone volume overestimations can possibly be solved by calibrating the measurements, provided that the degree of overestimation can be determined. If the purpose is to compare and analyse differences over time, this effect could be minimized by using the same CT device with a standardized imaging protocol.

Since the resolution of clinically available CT devices steadily increases, the results from this *in vitro* study suggest that ARG-based segmentation methods may also work for analyses of 3D trabecular bone microstructure in vivo. If conventional clinical CT devices could be used for follow-up and evaluation of bone structure changes in, for example, osteoporosis treatment, this would be an improvement for these patients.

## Conclusion

The strong correlations to micro-CT, support our hypothesis that an ARG-based segmentation code could be used for segmenting and measuring trabecular bone micro structure (TBMS) of HR-pQCT data of fresh-frozen human wrist specimens. Over- and under-estimation of the 3D bone structure parameters must however be taken into account.

## Authors´ contributions

Eva Klintström: Conceptualization, Methodology, Validation, Writing the original draft, Writing, Review and Editing, Supervision and Funding. Benjamin Klintström: Methodology, Software, Formal analysis, Investigation, Validation, Data curation, Visualization and Writing, Review and Editing. Örjan Smedby: Formal analysis and Writing, Review and Editing. Rodrigo Moreno: Software, Formal analysis and Writing, Review and Editing.

## Data Availability

All data and material can be made available after contact with the authors and the original data owners.
